# Venlafaxine Removal from Water and Wastewater Using Activated Carbons from Spent Brewery Grains Produced by Conventional vs. Microwave Pyrolysis

**DOI:** 10.3390/ph19030344

**Published:** 2026-02-24

**Authors:** Angelica R. Zizzamia, Ângela Almeida, María V. Gil, Filomena Lelario, Vânia Calisto

**Affiliations:** 1 Department of Basic and Applied Sciences, University of Basilicata, 85100 Potenza, Italy; angelicarebecca.zizzamia@unibas.it (A.R.Z.); filomena.lelario@unibas.it (F.L.); 2 Department of Chemistry and Centre for Environmental and Marine Studies (CESAM), University of Aveiro, 3810-193 Aveiro, Portugal; aaalmeida@ua.pt; 3 Instituto de Ciencia y Tecnología del Carbono (INCAR-CSIC), Francisco Pintado Fe 26, 33011 Oviedo, Spain; victoria.gil@incar.csic.es

**Keywords:** pharmaceuticals, pollution mitigation, adsorption, biomass, activated carbon, wastewater

## Abstract

**Background/Objectives:** The recent increase in antidepressant consumption, particularly venlafaxine, combined with the limited effectiveness of conventional wastewater treatment processes, has led to rising environmental concentrations. Adsorption methods have emerged as effective strategies for removing persistent pharmaceuticals without generating harmful by-products. This study aimed to develop and assess two activated carbons (ACs) derived from spent brewery grains as an efficient material for venlafaxine removal from wastewater. **Methods:** Two pyrolysis methods, conventional and microwave-assisted, were evaluated to assess their influence on the adsorption properties. The materials were characterized through nitrogen physisorption and scanning electron microscopy to evaluate surface area (*S*_BET_), porosity, and morphology. Their adsorption properties were examined through batch adsorption experiments to analyze kinetic and equilibrium behavior, and the efficacy was evaluated in both ultrapure water and real wastewater. **Results:** The obtained AC exhibited high porosity, with the *S*_BET_ ranging from 1080 to 1197 m^2^ g^−1^. Kinetic studies indicated that adsorption followed a pseudo-second-order model, achieving equilibrium within 2 h. The equilibrium data were optimally described by the Langmuir isotherm, indicating monolayer adsorption, with the maximum adsorption capacity of microwave-assisted AC reaching 74 ± 6 mg g^−1^. Microwave-assisted AC has shown higher efficiency than conventionally produced AC, demonstrating that this pyrolysis technique can produce materials with enhanced adsorption properties. **Conclusions:** This study evidences that microwave-assisted pyrolysis of an abundant agro-industrial residue yields high-performance materials capable of efficiently removing an antidepressant, included in the revised Urban Wastewater Treatment Directive, from complex effluents even at low doses, highlighting a sustainable route to mitigate pharmaceutical contamination in aquatic environments.

## 1. Introduction

Pharmaceuticals, considered contaminants of emerging concern, are increasingly recognized as a global environmental challenge [1,2,3]. Their widespread occurrence in aquatic environments is largely attributed to intensive human consumption and insufficient removal in conventional wastewater treatment plants (WWTPs) [4,5,6]. Among pharmaceuticals, the antidepressant venlafaxine (VFX), a serotonin–norepinephrine reuptake inhibitor (SNRI), has emerged as one of the most frequently detected contaminants in surface waters, with levels reaching up to 1000 ng L^−1^ [7,8] (the chemical structure of VFX is shown in the Supplementary Material (SM)) [9,10,11]. Pharmacokinetically, the majority of VFX (approximately 87% of the administered dose) is excreted unchanged in urine. VFX enters the environment primarily through human excretion and the discharge of untreated or partially treated wastewater [12], as well as via waste streams from hospitals, pharmaceutical production industries, and improper disposal of unused medicines [7]. In most cases, WWTPs are only able to eliminate about 30% of VFX [13,14], resulting in the discharge of contaminated wastewater into the aquatic environments. In fact, wastewater effluents in Europe, North America, and Asia have been found to present VFX concentrations ranging from ng L^−1^ to µg L^−1^ [13,14]. This occurrence, in addition to its pharmacological activity even at low concentrations, raises significant concerns regarding the potential impacts on aquatic ecosystems and, ultimately, on human health due to consumption of contaminated food and water. Its aqueous solubility is relatively high (572 mg/mL at 25 °C), contributing to its mobility and persistence in aquatic systems. Its log*K_ow_* value of 2.91 indicates moderate hydrophobicity and potential for bioaccumulation [7], which has been documented in species of fish from freshwater systems [15,16]. In addition, laboratory studies have revealed that environmentally relevant concentrations of VFX alter behavior, development, and endocrine function in aquatic organisms [17,18].

Due to widespread occurrence, incomplete removal on WWTPs, and ecotoxicological risks, VFX was first included in the 3rd Watch List (EU/2020/1161) [19] and retained in the 5th Watch List (EU/2025/439) [20]. In addition, in the revised version of the Urban Wastewater Directive ((UE) 2024/3019), VFX is one of the substances to be monitored in the wastewater effluents to assess successful contaminants removal [21]. Several advanced technologies have been reported in the literature for VFX removal from wastewater. Among them, adsorption is generally preferred for wastewater treatment for its low operational cost, simplicity, and minimal energy demand, making it suitable for both centralized and decentralized treatment systems [22,23]. Activated carbon (AC) is one of the most promising materials used in adsorption, exhibiting adequate surface properties (high surface area, well-developed porosity, and versatile surface chemistry) to ensure high efficiency even in complex aqueous matrices [7]. In addition, previous studies demonstrated that ACs can efficiently adsorb a wide range of pharmaceuticals, including VFX, with adsorption typically governed by pore filling and electrostatic interactions [7,24].

The performance of ACs strongly depends on their physicochemical properties, which, in turn, are influenced by the precursor material, activation process, and thermal treatment. To address sustainability concerns and reduce the environmental footprint of AC production, agro-industrial residues are increasingly used as precursors. Spent brewery grains (SBG), the primary by-product of the brewing industry, represent an abundant and underutilized lignocellulosic biomass, generated at an estimated rate of ~39 million tons per year worldwide [25]. Due to its high content of cellulose, hemicellulose, lignin, and proteins, SBG is a promising feedstock for producing high-performance carbon materials. Concerning thermal treatment, recent advances highlight the potential of microwave-assisted pyrolysis as a more efficient alternative to conventional pyrolysis for biomass valorization. Microwave-assisted pyrolysis enables rapid and homogeneous heating, leading to enhanced pore development and higher surface areas, while also reducing residence times and energy consumption [26,27,28].

This study aims to investigate the influence of two distinct pyrolysis approaches, conventional (CP) and microwave-assisted (MW) pyrolysis, on the synthesis and adsorption performance of ACs derived from SBG, namely, SBG-AC-CP and SBG-BC-MW, for the removal of VFX from aqueous solutions. Unlike previous studies that typically focus on single synthesis routes, this work systematically links the two different synthesis routes to textural/surface properties and adsorption performance in both water and wastewater. By integrating kinetic and equilibrium adsorption studies, it elucidates how the type of pyrolysis governs VFX adsorption efficiency, while contributing to the sustainable valorization of brewery residues into functional materials for water treatment applications.

## 2. Results and Discussion

### 2.1. Production and Characterization of Carbon Adsorbents

The production yield of the AC obtained by conventional (SBG-AC-CP) and microwave pyrolysis (SBG-AC-MP) was calculated using Equation (1). The yields obtained for SBG-AC-CP and SBG-AC-MP were approximately 7% and 3%, respectively. Among the processes involved in material production, the washing step inevitably caused material losses due to the removal of the inorganic fraction; this fraction is not of interest from the point of view of the adsorptive performance of the materials, leading to lower results per mass of adsorbent when it is not removed. The low yields for both materials may also be attributed to a substantial loss of volatile organic matter, resulting from the use of potassium carbonate (K_2_CO_3_) as a chemical activation agent. This activation agent effectively promoted high volatile release and development of a porous structure (as can be seen further from the *S*_BET_ analyses), which is highly recommended for the application considered in this study.

The point of zero charge (PZC) was determined to assess the net surface charge of the materials. A low PZC is generally associated with a high concentration of acidic surface groups. For SBG-AC-CP and SBG-AC-MP, the PZC values were quite acidic, around 4.0 and 3.5, respectively. This acidity is likely due to chemical activation with K_2_CO_3_, due to the incorporation of oxygen-containing functional groups into the surface of the materials. In contrast, Sousa et al. [29] reported neutral (6.9) or slightly basic PZC (8.0) values for ACs prepared from SBG via conventional pyrolysis and chemical activation with potassium hydroxide (KOH) instead of K_2_CO_3_.

Since the surface charge of ACs depends on both the solution pH and their PZC, in aqueous solutions with pH below the PZC, the surface is predominantly positively charged, whereas for pH above the PZC, it becomes predominantly negatively charged. At the working pH values of the matrices selected in this study (approximately 6 for ultrapure water and 7.7 for wastewater), the surface of both materials is therefore mainly negatively charged, and the observed differences in the PZC between the two materials are not expected to significantly impact the adsorptive performance of the materials in the context under study.

Regarding the FTIR-ATR analyses, the spectra of the precursor (SBG) and the produced activated carbons (SBG-AC-CP and SBG-AC-MW) are presented in Figure S1 of the Supplementary Material. The spectra clearly show that SBG exhibits a more complex chemical composition compared to the derived ACs. In the spectrum of SBG, the bands observed at 2935–2915 cm^−1^ and 2865–2845 cm^−1^ are attributed to asymmetric and symmetric C–H stretching vibrations of aliphatic –CH_2_ and –CH_3_ groups, respectively [30]. These functional groups are characteristic of lignocellulosic components such as cellulose, hemicellulose, and lignin [31]. A weaker contribution in the 2880–2860 cm^−1^ region may also be associated with aliphatic C–H stretching vibrations. In SBG-AC-CP and SBG-AC-MW, these bands are significantly reduced or absent, indicating the decomposition of aliphatic structures during the thermal degradation (pyrolysis) process. The distinct band at approximately 1743 cm^−1^, present in SBG, corresponds to C=O stretching vibrations of carbonyl-containing groups such as ketones, aldehydes, esters, and carboxylic acids [32]. The reduction or modification of this band in the ACs reflects structural rearrangements and partial decomposition of oxygenated functionalities during pyrolysis. However, the presence of carbonyl-related bands in the ACs suggests that some oxygenated groups remain or are formed during activation with potassium carbonate. The signals in the 1617–1641 cm^−1^ region are attributed to aromatic C=C stretching vibrations, indicating the formation of conjugated graphitic domains within the carbon matrix [32]. This increase in aromatic character is typical of thermochemically treated lignocellulosic precursors and confirms the progressive carbonization of the biomass structure.

Differences between SBG-AC-MP and SBG-AC-CP indicate that the pyrolysis method influences the surface chemistry and distribution of functional groups. Bands observed between 1238 and 1026 cm^−1^ are assigned to C–O stretching vibrations of phenolic, alcoholic, ether, and carboxylic groups [33,34]. The band near 1153 cm^−1^ is associated with C–O–C stretching of cellulosic ethers, the peak around 1238 cm^−1^ corresponds to C–O stretching in carbonyl-related structures [30]. The band at approximately 1394 cm^−1^ is attributed to C–H bending vibrations [35], and the signal near 1641 cm^−1^ may also be associated with conjugated carbonyl groups within aromatic lignin-derived structures [36]. The band around 1009–1025 cm^−1^ is attributed to C–O stretching vibrations of carboxylic groups [37]. Additionally, the peaks observed in the 980–900 cm^−1^ region, particularly in the ACs, are associated with C–H out-of-plane bending vibrations of aromatic rings, further supporting the development of condensed aromatic structures after pyrolysis.

Overall, the FTIR results confirm that pyrolysis and chemical activation significantly altered the chemical structure of SBG, promoting the degradation of aliphatic components and enhancing aromaticity while preserving or generating oxygen-containing surface functional groups that are relevant for adsorption processes.

To investigate the textural features of the materials produced, nitrogen adsorption isotherms were carried out. The main textural parameters of the produced materials, including *S*_BET_, total pore volume (*V_p_*), micropore volume (*W*_0_), average micropore width (*L*), and average pore diameter (*D*), are presented in Table 1.

The *S*_BET_ values for SBG-AC-CP and SBG-AC-MP were 1080 and 1197 m^2^ g^−1^, respectively. A similar trend was observed for the total pore volume (*V*_p_) and micropore volume (*W*_0_), both of which, although slightly, were higher for SBG-AC-MP than for SBG-AC-CP. These textural characteristics are expected to positively influence the adsorptive capacity of the materials. In fact, the results indicate that microwave-assisted pyrolysis enhances the development of microporosity to a greater extent than conventional pyrolysis, as it promotes faster, more homogeneous heating of the precursor material, thereby facilitating the formation of a microporous network. The data obtained in this work are consistent with findings from other studies, such as that of Sousa et al. [29], in which ACs were produced from conventional pyrolysis of SBG preceded by chemical activation with KOH, sodium hydroxide (NaOH), or phosphoric acid (H_3_PO_4_). The authors tested different pyrolysis temperatures and residence times; at 800 °C and a 150 min residence time, the *S*_BET_ values obtained with KOH, NaOH, and H_3_PO_4_ activation were 1090 m^2^ g^−1^, 18 m^2^ g^−1^, and 14 m^2^ g^−1^, respectively [29]. Only the KOH-activated AC exhibited an *S*_BET_ (1090 m^2^ g^−1^) comparable to those of the ACs produced in this work (1080 m^2^ g^−1^ for SBG-AC-CP and 1197 m^2^ g^−1^ for SBG-AC-MP). The formation of microporosity in ACs derived from SBG has also been reported in previous studies [38]. Sieradzka et al. [38] demonstrated that the brewery grains precursor in its raw form, and the biochar obtained by conventional pyrolysis at 400 °C with and without KOH chemical activation, exhibited *S*_BET_ values of 0.36 m^2^ g^−1^, 0.67 m^2^ g^−1^, and 2619 m^2^ g^−1^, respectively. These results highlight the crucial role of chemical activation in producing ACs with high *S*_BET_, which, in turn, ensures good adsorption performance and favorable removal efficiencies. However, this high *S*_BET_ is generally achieved with two-step pyrolysis (pyrolysis and then impregnation with an activating agent, followed by a second pyrolysis), with a high impact on the energy consumption during the process [38]. In the present work, milder conditions were used, seeking a more sustainable approach: the use of one-step microwave-assisted pyrolysis. In other studies, such as that by Fontana et al. [39], SBG was used in its raw form—merely dried, crushed, and sieved—resulting in an *S*_BET_ of 0.82 m^2^ g^−1^. This further emphasizes that the precursor by itself (without chemical activation and thermal treatment) is insufficient to yield a material with the properties of an effective adsorbent. Gómez-Delgado et al. [40] developed ACs from SBG for the removal of azo dye Orange II. The precursor was treated with NaOH at different mass ratios (1:1 up to 3:1) and activated at temperatures from 400 to 600 °C through conventional pyrolysis. The resulting materials achieved *S*_BET_ surface areas ranging from 3.46 to 466 m^2^ g^−1^ [40].

**Figure 1:**
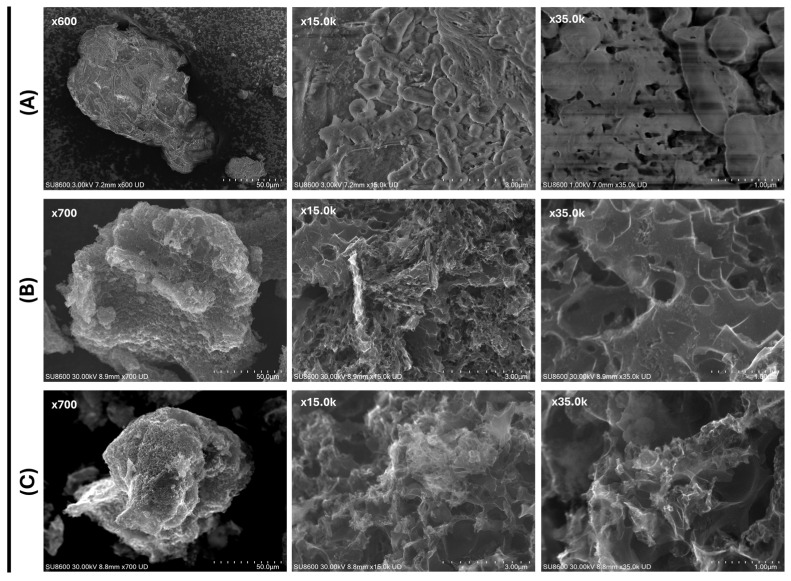
SEM images of (**A**) the precursor SBG at magnifications of ×600, ×15.0 k, and ×35.0 k, (**B**) SBG-AC-CP, and (**C**) SBG-AC-MP at magnifications of ×700, ×15.0 k, and ×35.0 k.

Scanning Electron Microscopy (SEM) images obtained for the precursor, SBG-AC-CP, and SBG-AC-MP (Figure 1) reveal clear differences in surface morphology between them. The surface of the precursor SBG appears homogeneous and smooth, with no evident porous structure (Figure 1A). In contrast, both ACs display much rougher surfaces with well-developed porosity (Figure 1B,C), which is favorable for effective adsorbent–adsorbate interactions, as confirmed by the high *S*_BET_, *V*_p_, and *W*_0_ values reported in Table 1.

### 2.2. Adsorption Experiments

#### 2.2.1. Preliminary Tests

Preliminary tests were carried out both in ultrapure water and wastewater. Physicochemical parameters were measured for the collected wastewater, including pH (7.7 ± 0.1), conductivity (2.7 mS cm^−1^), resistivity (0.0004 MΩ·cm), salinity (1.4 PSU), dissolved oxygen (7.8 mg L^−1^), and redox potential (38.9 mVORP) at 21.9 °C. The measured total organic carbon (TOC) was 12.8 ± 0.3 mg L^−1^.

**Figure 2:**
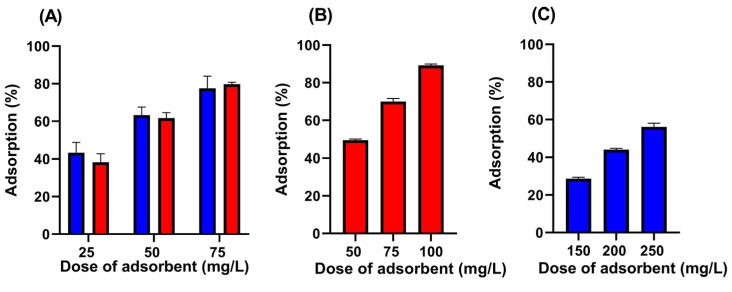
Adsorption percentages of VFX (5 mg L^−1^) in ultrapure water (**A**) and in wastewater (**B**) and (**C**) at different doses of adsorbent materials, using SBG-AC-CP (blue) and SBG-AC-MP (red). Each bar represents the mean ± standard deviation (n = 3).

Figure 2 shows the histogram-like plot of the adsorption percentage of VFX onto two SBG-AC-CP and SBG-AC-MP in ultrapure water and wastewater, at different material doses. Both SBG-AC-CP and SBG-AC-MP demonstrated high performance even at very low adsorbent doses, as shown in Figure 2.

After 24 h of contact between the adsorbent and VFX, in ultrapure water, the percentage removal was 63 ± 4% for SBG-AC-CP and 62 ± 3% for SBG-AC-MP, using an adsorbent dose of only 50 mg L^−1^ (Figure 2A). The two materials exhibited equivalent adsorptive performance in VFX removal from ultrapure water (no significant difference at 95% confidence level). In particular, the presence of hydroxyl and methoxy substituents in VFX’s structure enables hydrogen bonding with oxygen-containing surface groups (carboxyl, hydroxyl, carbonyl) on the adsorbent. At the same time, π–π interactions can occur between the aromatic moiety of VFX and the graphitic domains of carbon materials, further enhancing adsorption [7]. Thus, the adsorption mechanism of VFX is typically governed by a combination of hydrophobic partitioning, electrostatic interactions, hydrogen bonding, and π–π stacking [41]. For both materials, a dose of 50 mg L^−1^ was selected for subsequent kinetic tests in ultrapure water.

Wastewater was also used for the preliminary evaluation of the adsorptive performance of the two materials under more realistic and representative conditions. To assess the adsorption capacity of the materials in wastewater, three adsorbent doses (150, 200, and 250 mg L^−1^), higher than those used in ultrapure water (25, 50, and 75 mg L^−1^), were applied, as the competitive and inhibitory effects of the real matrix must be considered, which may reduce the adsorption capacity of the materials. For SBG-AC-MP, complete removal of VFX was achieved at all three doses (below the limit of detection), rendering the drug peak undetectable by HPLC-FLD (High-Performance Liquid Chromatography with Fluorescence Detection). Consequently, the doses for SBG-AC-MP were subsequently reduced to 50, 75, and 100 mg L^−1^. The adsorption percentages of the two materials for VFX in wastewater differed markedly (Figure 2): in both cases, the removal efficiency increased as the adsorbent dose increased, but for SBG-AC-MP, a dose of 50 mg L^−1^ was sufficient to achieve ~50% VFX removal, whereas SBG-AC-CP required about fivefold higher doses. Thus, although the adsorption performance is similar in ultrapure water, when comparing the two materials in wastewater, the performance gap becomes evident, highlighting the importance of testing relevant matrices for the target application, as extrapolation of results can lead to erroneous conclusions.

Comparing removal percentages in ultrapure water and wastewater, SBG-AC-MP achieved, respectively, 62 ± 3% and 49.5 ± 0.6% VFX removal using the same dose of 50 mg L^−1^ (Figure 2A,B, red bars). The lower removal in wastewater is due to matrix effects. A statistical comparison of these data (*t*-test, at 95% confidence level) revealed that SBG-AC-MP exhibited statistically different adsorptive performance in VFX removal from ultrapure water and wastewater. Despite the statistical difference, the values are quite close; yet, the situation is different for SBG-AC-CP: with a dose of 50 mg L^−1^, a removal of 63 ± 4% is achieved, but an adsorbent dose of at least 200 mg L^−1^ was required to reach more than 40% VFX removal in wastewater (Figure 2A,C, blue bars). These results highlight the better adsorption capacity of SBG-AC-MP and its superior robustness under realistic conditions.

As previously referred, differences in adsorption between wastewater and ultrapure water might be affected by inhibitory and matrix effects, as well as the interplay between VFX protonation and the materials’ PZC. Because VFX has a pKa of 9.6, the Henderson–Hasselbalch equation indicates that, both at ultrapure water (pH around 6) and wastewater (pH 7.7), VFX will be predominantly in its protonated form (more than 98% speciated in the positively charged form), enabling strong electrostatic attraction with the negatively charged AC surfaces. Therefore, the significant decrease in the adsorption capacity of SBG-AC-CP from ultrapure water to wastewater cannot be explained by differences in electrostatic interactions in this particular case, as pore blockage by matrix components is the most likely explanation for the results obtained. Moreover, common inorganic ions, such as chloride (Cl^−^), sulfate (SO_4_^2−^), and calcium (Ca^2+^), may also interfere through multiple mechanisms. These ions can compete with the target contaminant for adsorption sites, contribute to increased ionic strength that weakens electrostatic interactions, and, in the case of multivalent ions, promote partial pore blockage [42]. Together with dissolved organic matter, these effects can lead to lower adsorption efficiencies in wastewater than in ultrapure water, even when contaminant speciation remains essentially unchanged and should therefore be considered when assessing the practical applicability of the materials [43].

Overall, SBG-AC-CP showed poor performance in wastewater, the target application of this adsorptive process, and, combined with its harsher preparation conditions, makes it a less promising material than SBG-AC-MP. The decline in efficiency is less severe for SBG-AC-MP, which performs effectively even under matrix-limited conditions. Thus, for this reason, further kinetic and equilibrium studies in wastewater were carried out only for SBG-AC-MP, as it showed the most promising material in terms of both adsorption performance and energy-efficient production.

#### 2.2.2. Adsorption Kinetics

**Figure 3:**
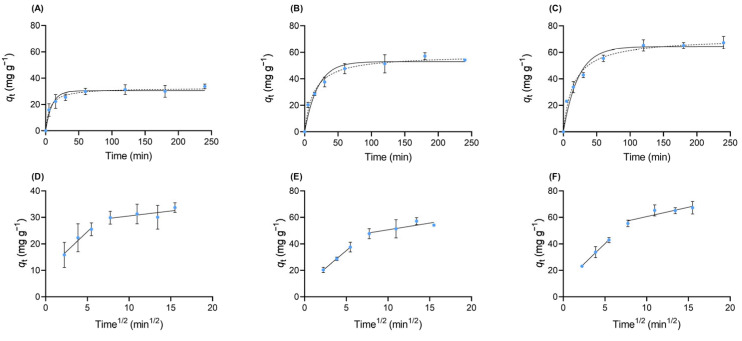
Kinetic fittings for the experimental data of VFX adsorption onto SBG-AC-CP in ultrapure water in (**A**,**D**), SBG-AC-MP in ultrapure water in (**B**,**E**), and SBG-AC-MP in wastewater in (**C**,**F**). The results were fitted to the pseudo-first-order (solid lines on (**A**–**C**)), pseudo-second-order (dashed lines on (**A**–**C**)), and the Weber–Morris intraparticle diffusion model (on (**D**–**F**)) kinetic models. Each data point (±standard deviation) represents the mean of three replicates. Experimental conditions: VFX concentration 5 mg L^−1^; adsorbent dose 50 mg L^−1^; stirring at 80 rpm; temperature 25.0 ± 0.1 °C.

Based on the preliminary tests (Section 2.2.1), a dose of 50 mg L^−1^ was selected for all kinetic experiments. To determine the time required to reach adsorption equilibrium of VFX onto SBG-AC-MP, kinetic studies were conducted in ultrapure water and wastewater, and onto SBG-AC-CP in ultrapure water. The graphical representation of the experimental kinetic results (*q_t_* (mg g^−1^) vs. time (min)) is shown in Figure 3, together with the fitting curves of the applied mathematical models (Equations (4)–(6), Section 3.4.2). The kinetic data for VFX adsorption onto SBG-AC-MP and SBG-AC-CP were fitted to the pseudo-first-order, pseudo-second-order, and Weber–Morris intraparticle diffusion models.

Table 2 summarizes the kinetic parameters obtained from fitting the experimental data to the pseudo-first-order, pseudo-second-order, and intra-particle diffusion models for VFX adsorption onto SBG-AC-CP and SBG-AC-MP in ultrapure water and wastewater.

The comparison of kinetic profiles between SBG-AC-CP and SBG-AC-MP, as well as between their performance in ultrapure water and wastewater systems, enables the assessment of both the production process and the solution matrix on the adsorption rate. Across all systems, the pseudo-second-order model yielded a superior fit, as indicated by higher determination coefficients (*r*^2^) and lower residual errors *S_y/x_* (Table 2). Although the pseudo-first-order model presents *r*^2^ values above 0.95 for all experimental conditions, it less accurately represented the data compared with the pseudo-second-order model (*r*^2^> 0.98). This was further evidenced by the higher *S_y/x_* values (Table 2) obtained for the pseudo-first-order fitting (2.584, 4.387, and 5.193) relative to those for the pseudo-second-order fitting (1.332, 2.663, and 3.092). Consequently, the pseudo-second-order model was selected for further discussion. According to this model, the equilibrium adsorption capacities (*q*_e_) for VFX removal were 32.6 ± 0.8 mg g^−1^ for SBG-AC-CP in ultrapure water, 58 ± 2 mg g^−1^ for SBG-AC-MP in ultrapure water, and 71 ± 2 mg g^−1^ for SBG-AC-MP in wastewater, all obtained under the same conditions, including material dose. As shown in Figure 3A–C and Table 2, VFX adsorption onto both materials proceeded relatively rapidly, with equilibrium reached in 1 h. In all cases, a steep increase in *q*_t_ values was observed during the first ~30 min, in both ultrapure water and wastewater, indicating a fast initial uptake phase driven by the abundance of readily accessible active sites on the adsorbent surface. SBG-AC-CP in ultrapure water exhibited a slower approach to equilibrium and a lower *q*_e_ compared with SBG-AC-MP, underscoring the role of preparation method and resulting textural properties in determining adsorption performance of chosen materials.

Fittings to the Weber–Morris intraparticle diffusion model for the experimental kinetic data obtained in ultrapure water and wastewater were also determined and are shown in Figure 3D–F. All systems exhibit multi-linear behaviour with two distinct stages: the initial steeper phase (higher k_id(1)_) corresponds to external film diffusion, while the second phase (lower k_id(2)_) reflects intraparticle diffusion within the AC pores. In all the studied systems, it is evident that there is a significant decrease in slope from the first to the second stage, which indicates progressively slower diffusion as pores fill, typical of microporous adsorbents. For SBG-AC-MP, the comparable k_id_ values across both matrices (ultrapure water vs. wastewater) demonstrate efficient mass transfer throughout the process, with the enhanced microporosity from microwave pyrolysis facilitating VFX access even under matrix interference. The similar intercepts (C) in phase 1 for both matrices suggest that wastewater components did not substantially increase external mass transfer resistance compared to ultrapure water, further evidencing SBG-AC-MP robustness.

The adsorption kinetics obtained in this study for VFX are fully aligned with those reported by other works [44,45,46,47]. Table 3 summarizes literature data on the adsorption kinetics of various organic contaminants from ACs derived from SBG, reporting pseudo-first-order and pseudo-second-order rate constants, correlation coefficients, and best-fit models, and provides a direct comparison with the findings of the present study. The studies in Table 3, involving structurally diverse contaminants, including pharmaceuticals such as acetaminophen, sulfamethoxazole, trimethoprim, ciprofloxacin, and carbamazepine, and the dye tartrazine, consistently indicate that adsorption onto AC is better described by the pseudo-second-order kinetic model, with reported correlation coefficients generally exceeding 0.94, except for ciprofloxacin [45,46,47,48,49]. In addition, *k*_2_ values remain relatively low in the 10^−3^–10^−4^ g·mg^−1^·min^−1^ range across adsorbent doses between 15 and 1000 mg L^−1^. In addition, concerning the type of pyrolysis, the majority applied hydrothermal or microwave-assisted processes for the carbonization of SBG, except Castro et al. [44], who evaluated conventional pyrolysis, generating adsorbents with lower *S*_BET_, requiring higher doses of material (200 mg L^−1^) for the removal of the yellow dye. The overall findings obtained in the present study provided the basis for the subsequent equilibrium studies, aimed at quantifying the maximum adsorption capacities.

#### 2.2.3. Adsorption Equilibrium

**Figure 4:**
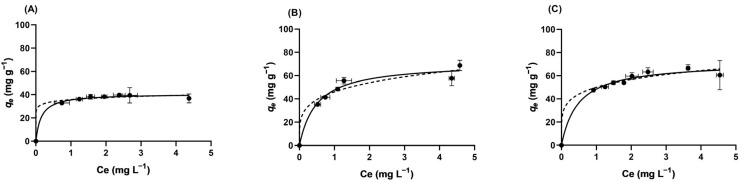
Equilibrium adsorption for the experimental data of VFX adsorption onto SBG-AC-CP in ultrapure water (**A**), SBG-AC-MP in ultrapure water (**B**), and SBG-AC-MP in wastewater (**C**). The experimental data were fitted to both the Langmuir (solid lines) and the Freundlich (dashed lines) models. Each experimental point represents the mean of three replicates (±standard deviation). Conditions: VFX at an initial concentration of 5 mg L^−1^, adsorbent doses 5–125 mg L^−1^, stirring (80 rpm), at 25.0 ± 0.1 °C.

Following the kinetic evaluation (Section 2.2.2), equilibrium adsorption studies were carried out to determine the maximum adsorption capacities of the produced ACs and to elucidate the influence of solution chemistry on VFX uptake. In fact, the adsorption isotherms represented the amount of VFX adsorbed onto SBG-AC-CP and SBG-AC-MP at equilibrium (*q_e_*, mg g^−1^) versus the amount of pharmaceutical remaining in solution (*C*_e_, mg L^−1^). These experiments allow for a deeper understanding of the interaction between the adsorbent surface and the VFX under varying conditions, complementing the kinetic analysis and providing essential parameters for practical application. The experimental equilibrium data were fitted to the Langmuir and the Freundlich isotherm models (Equations (7) and (8), Section 3.4.3). The Langmuir model assumes monolayer adsorption onto a homogeneous surface with a finite number of identical sites, whereas the Freundlich model is an empirical equation that accounts for multilayer adsorption on heterogeneous surfaces. Figure 4 shows the experimental equilibrium data and the corresponding Langmuir and Freundlich isotherm fits for VFX adsorption onto SBG-AC-CP and SBG-AC-MP in ultrapure water and wastewater.

Table 4 reports the fitting parameters obtained from the Langmuir and the Freundlich isotherm models for VFX adsorption onto SBG-AC-CP and SBG-AC-MP in ultrapure water and wastewater. These parameters allow comparison of the maximum adsorption capacity and the affinity of the adsorbent for VFX under different conditions, as well as the assessment of which model best describes the adsorption process.

Overall, as indicated by the *r*^2^ and *S_y/x_* values reported in Table 4**,** the equilibrium experimental data were well fitted by both the Langmuir and the Freundlich models (*r*^2^ ≥ 0.95). However, the Langmuir model showed slightly higher *r*^2^ values and lower *S_y/x_*, suggesting a better fit. A clearer difference was observed for VFX adsorption onto SBG-AC-MP in ultrapure water, where the Langmuir model yielded *r*^2^ = 0.9725 and *S_y/x_*= 4.048, compared to *r*^2^ = 0.9560 and *S_y/x_*= 5.118 for the Freundlich model. This indicates that in this specific case, the Langmuir equation provides a noticeably better description of the equilibrium data, highlighting that VFX adsorption onto SBG-AC-CP and SBG-AC-MP occurs predominantly as a monolayer on a homogeneous surface with uniform adsorption sites. Consequently, the Langmuir model fitting was adopted for the subsequent discussion and interpretation of adsorption capacity parameters. The results clearly show that SBG-AC-CP performs significantly worse than SBG-AC-MP in ultrapure water, with the latter exhibiting a 73% higher maximum adsorption capacity (41 ± 1 mg g^−1^ versus 71 ± 4 mg g^−1^). Statistical analysis confirms the significant difference between the two materials at the 95% confidence level (Table 4). Moreover, no difference was found in the maximum adsorption capacity of SBG-AC-MP between ultrapure water (71 ± 4 mg g^−1^) and wastewater (72 ± 3 mg g^−1^) at the 95% confidence level, which constitutes a remarkable feature of this material, confirming that the complexity of the matrix does not adversely affect its adsorptive properties. These findings, combined with the kinetic results, suggest that the enhanced performance of SBG-AC-MP might be linked to its higher *S*_BET_ and slightly larger *V*_p,_ which promote more efficient access to adsorption sites. Thus, for this material, competitive adsorption in wastewater has a negligible effect on the adsorption capacity towards VFX. In addition, as referred in Section 2.2.1, electrostatic interactions do not play a relevant role in the VFX uptake from the studied matrices due to the acidic nature of the material combined with the weak base properties of VFX. In fact, the maximum adsorption capacity of SBG-AC-MP in wastewater remains higher than that of SBG-AC-CP in ultrapure water (72 ± 3 mg g^−1^ vs. 41 ± 1 mg g^−1^). This finding highlights not only the intrinsic superiority of the microwave-derived AC but also its robustness under realistic environmental conditions. As discussed in Section 2.1, the unique advantages of microwave pyrolysis over conventional pyrolysis lie in its volumetric heating mechanism, which overcomes the heat/mass transfer limitations of conductive heating. In fact, microwave-assisted pyrolysis promotes the formation of a greater number of micropores, leading to increased textural properties compared to conventional pyrolysis, even when the same chemical activating agent is used. Moreover, microwave pyrolysis offers additional benefits such as shorter processing times and lower energy consumption, making it a more sustainable and efficient approach to AC production. In the specific case of this study, microwave-assisted pyrolysis can be correlated to (i) greater microporosity development (32% improvement in *W*_0_ compared with conventional pyrolysis); (ii) shorter residence times (20 versus 120 min), reducing energy consumption; and (iii) lower yields but higher adsorbent efficiency per gram of the material, as evidenced by the results presented in this sub-section. Consequently, SBG-AC-MP achieves comparable or better performance than the CP material at much lower doses in wastewater (5× lower), highlighting microwave pyrolysis as a viable route for high-value AC production from agro-residues.

The comparative evaluation of equilibrium parameters obtained in this study and those reported in the literature further highlights differences in adsorption performance that arise from variations in pyrolysis method, activation chemistry, and the physicochemical characteristics of each adsorbate [45,46,47,48,49]. Table 3 presents the adsorption isotherm parameters for VFX onto SBG-AC-MP, along with data for other pharmaceuticals adsorbed onto various SBG-derived activated carbons reported in the literature.

The equilibrium adsorption behavior of SBG-derived activated carbons shows that the nature of the adsorbate and solution conditions strongly influence adsorption capacity. In the study by Castro et al. [44], tartrazine yellow dye exhibited a maximum adsorption capacity of 32 mg g^−1^ on SBG-derived AC at pH 3, with equilibrium data well described by the Langmuir isotherm. In the work of Araújo et al. [45], the activated hydrochar exhibited a very high maximum adsorption capacity (*q*_m_ = 318 mg g^−1^) toward acetaminophen, with the equilibrium data fitting the Langmuir isotherm, indicating monolayer adsorption on uniform active sites. This high *q*_m_ (318 mg g^−1^) is primarily attributed to the exceptionally large surface area of the hydrochar (1513 m^2^ g^−1^) generated through hydrothermal carbonization followed by KOH activation, as well as favorable interactions between acetaminophen molecules and surface functional groups [45]. The combination of a high activating agent/precursor ratio (4:1) and an intense hydrothermal process improves adsorption, but at the cost of higher processing costs and reduced sustainability. In contrast, the SBG-AC-MP developed in this work was produced via microwave-assisted pyrolysis with a lower K_2_CO_3_/precursor ratio (1:2), resulting in a moderately high surface area and a *q*_m_ of 71 mg g^−1^ for VFX. Although this activation is less aggressive than hydrothermal KOH treatment, it still generates sufficient porosity and surface functionality for effective adsorption at environmentally relevant pH and at very low doses of material. These results underscore that both the activating agent/precursor ratio and the type of thermal treatment directly influence carbon structure and adsorption performance, with more intensive conditions yielding higher-performing materials, albeit with increased cost and synthesis complexity. In the work by Sousa et al. [46], sulfamethoxazole, trimethoprim, and ciprofloxacin were adsorbed onto microwave-assisted carbons obtained using the same procedure as in the present work. These ACs exhibited lower *S*_BET_ (929 m^2^ g^−1^) compared to those of the present work’s ACs (1197 m^2^ g^−1^); the higher adsorption capacities (*q*_m_ = 120, 244, and 116 mg g^−1^ for sulfamethoxazole, trimethoprim, and ciprofloxacin, respectively), obtained by Sousa et al. [46], are likely due to operating conditions and a stronger interaction affinity with the adsorbent surface. In another study by Apinyakul et al. [47], carbamazepine adsorption onto hydrothermally produced carbon (activated by 1:1 KOH:NaCl, *w/w*) at pH 7, with *S*_BET_ = 906 m^2^ g^−1^, resulted in a much larger maximum adsorption capacity than the ones reported in this study; yet a 60-times higher adsorbate concentration was used along with a material dose 20 times higher than the one tested in this work [47]. This discrepancy in adsorption conditions highly affects the determined performance and must be carefully considered, as it hinders a direct comparison of results obtained in such distinct conditions.

In addition to the clear impact on the textural properties and adsorption performance of the resulting materials (Table 3), the choice of activating agent has important environmental and operational implications. Several studies have highlighted that KOH, although highly effective at generating very high surface areas, is a strongly corrosive reagent that produces large volumes of highly alkaline washing effluents, requiring careful neutralization and increasing corrosion risks in equipment, complicating large-scale applications [49]. By contrast, K_2_CO_3_ is a milder, less corrosive reagent that yields activation liquors that are easier to neutralize and handle and is therefore regarded as a greener and safer alternative for the chemical activation of biomass-derived carbons [50]. Also, some comparative literature studies applying both agents evidence that for optimized conditions, K_2_CO_3_-activated materials can achieve similar textural properties to KOH-activated materials [29], further supporting the adequacy of this activating agent. In this context, the use of K_2_CO_3_ in the present work is consistent with recent efforts to replace harsh activating agents with more sustainable options without compromising adsorption performance.

Overall, the equilibrium adsorption study confirmed the high efficiency of SBG-AC-MP in removing VFX, while the similar *q*_m_ values in ultrapure water and wastewater highlight its robustness under realistic conditions. The Langmuir model provided the best fit, indicating monolayer adsorption on homogeneous sites. Combined with kinetic results, these findings demonstrate that microwave-assisted pyrolysis with K_2_CO_3_ activation yields an adsorbent with superior textural and adsorptive properties compared to conventional pyrolysis, enabling high efficiency, even in complex wastewater.

Overall, the results discussed here demonstrate encouraging adsorption performance of SBG-AC-MP at the laboratory scale; however, comprehensive investigations are still required to determine its economic viability for real-world wastewater treatment applications. Future research should therefore focus on designing pilot-scale experiments that support a robust techno-economic evaluation encompassing production at industrial furnaces, possible regeneration, and large-scale deployment costs.

## 3. Materials and Methods

### 3.1. Reagents and Chemicals

The reagent used in the chemical activation process was potassium carbonate (K_2_CO_3_, AnalaR normapur, 99.9%). For washing the produced material, hydrochloric acid (HCl, Honeywell FLUKA, 37.0%) was employed. HCl, sodium hydroxide (NaOH, José Manuel Gomes dos Santos, 99.3%), and sodium chloride (NaCl, Honeywell FLUKA, ≥99.5%) were used to determine the point of zero charge (PZC).

Venlafaxine hydrochloride (VFX, TCI, 98%) was the pharmaceutical used for adsorption experiments. Physico-chemical properties of VFX, along with its chemical structure, can be found in Table S1 in the Supplementary Material. For chromatographic analyses, acetonitrile and methanol (both HPLC grade) were obtained from Fisher Scientific, while HCl (37%) was supplied by Honeywell FLUKA, as previously referred. Ultrapure water was sourced from an Elga Purelab Flex 4 purification system (Veolia, ELGA LabWater, United Kingdom).

### 3.2. Carbon Adsorbents Production and Characterization

SBG was collected from Brewery Faustino Microcervejeira, Lda (Aveiro, Portugal), dried at room temperature, and then dried for an additional 24 h at 100 °C. Then, 570 g of SBG was ground using a blade mill and impregnated with K_2_CO_3_ at an activating agent/SBG 1:2 mass ratio, using a 0.15 g mL^−1^ solution of K_2_CO_3_. The mixture was then stirred in an ultrasonic bath for 1 h and left to dry at room temperature for several days. A portion of the dried impregnated material was carbonized under a nitrogen atmosphere in a muffle furnace (Nüve, series MF 110, Turkey). The activated precursor was heated at a rate of 10 °C min^−1^ until it reached 800 °C, with a 2 h residence time at this temperature, resulting in the SBG-AC-CP material. The other portion was carbonized in a microwave furnace (CEM Phoenix™ AirWave, Germany) at 800 °C (heating rate of 15 °C min^−1^), with a 20 min residence time, resulting in the material named as SBG-AC-MP. The resulting materials were washed with approximately 1.2 M HCl, followed by distilled water, until the leachate reached a pH around 6. Finally, the produced SBG-AC-CP and SBG-AC-MP were dried overnight at 50 °C, crushed, and sieved to obtain a fine powder with a particle size ≤ 180 μm. The materials were stored in a desiccator to ensure dryness and preserve their physicochemical stability.

The yield of production (*η*) of both carbon adsorbents produced was calculated by Equation (1):

(1) $$\eta \;(\%)\;=\;\frac{\mathrm{final}\;\mathrm{mass}\;\mathrm{of}\;\mathrm{carbon}\;\mathrm{adsorbent}\;\left(g\right)}{\mathrm{mass}\;\mathrm{of}\;\mathrm{precursor}\;\left(g\right)}\times 100$$

Both materials were characterized regarding surface/textural properties and applied in the removal of VFX, using batch adsorption studies for the evaluation of kinetic and equilibrium system parameters, as described in the next sections.

Both SBG-AC-CP and SBG-AC-MP samples were characterized by Point of Zero Charge (PZC) determination, Scanning Electron Microscopy (SEM), Fourier transform infrared spectroscopy with attenuated total reflectance (FTIR-ATR), and N_2_ adsorption isotherms to determine the specific surface area (*S*_BET_) and pore distribution. The description of these analyses is detailed in the Supplementary Material (SM).

### 3.3. Wastewater Sampling

Wastewater samples were collected in June 2025 from a local urban WWTP in Aveiro, Portugal. The samples corresponded to the final effluent, obtained after biological treatment. Immediately after collection, to remove suspended organic matter, the effluent was filtered through 0.45 μm cellulose Supor-450 membrane disc filters using a vacuum system. The filtered samples were stored in the dark at 4 °C and used within 15 days. The effluent was characterized by measuring pH with a Hanna Instruments HI2020-02 pH meter (Romania). Other physicochemical parameters were also measured for the collected wastewater, such as conductivity, resistivity, salinity, dissolved oxygen, and redox potential using a multiparameter sensor (HI98594, Hanna instruments, Romania). The total organic carbon (TOC) was determined using a Shimadzu TOC-VCPH liquid sample module SSM-5000 A (Japan), carried out in triplicate.

### 3.4. Adsorption Experiments

#### 3.4.1. Preliminary Tests

To evaluate the adsorptive performance of the produced materials for VFX removal from ultrapure water and wastewater, batch adsorption experiments were conducted under controlled temperature and shaking conditions. In a preliminary approach, adsorption tests were performed to determine the optimal dose of each material for subsequent kinetic and isothermal studies. A VFX solution with an initial concentration of 5 mg L^−1^ was prepared in ultrapure water and distributed into polypropylene tubes containing three different adsorbent masses, corresponding to final doses of 25, 50, and 75 mg L^−1^. Samples were stirred in an overhead shaker (Heidolph Reax 2, Germany) at 80 rpm for 24 h at 25.0 ± 0.1 °C. Additionally, batch adsorption experiments in wastewater were carried out to evaluate matrix and competitive effects in real effluents on the adsorptive performance of two materials in the removal of the antidepressant drug. Preliminary tests in wastewater were carried out in the same way as those in ultrapure water, using higher doses of adsorbents (150, 200, and 250 mg L^−1^).

Following shaking, aliquots of the samples were filtered through 0.22 μm PVDF filters (Whatman, Cytiva, United Kingdom) and analyzed by HPLC-FLD, as described in Section 3.5. Based on the adsorption percentages obtained in the preliminary test, the adsorbent dose yielding 40–60% removal of VFX was selected for further kinetic experiments, to ensure meaningful adsorption combined with feasible analysis of this antidepressant in the aqueous phase. All tests (preliminary, kinetic, and equilibrium) were performed in triplicate, with pharmaceutical solution controls (without adsorbent) used as references for adsorption percentage calculations according to Equation (2):

(2) $$\mathrm{Adsorption}\;(\%)\;=\;\frac{C_{c}-C_{f}}{C_{c}}\times \;100$$

where *C*_c_ (mg L^−1^) is the concentration of pharmaceutical in the corresponding control experiments (from now on, taken as the initial pharmaceutical concentration) and *C*_f_ (mg L^−1^) is the remaining pharmaceutical concentration in the liquid phase at the end of the adsorption experiment with each material.

#### 3.4.2. Adsorption Kinetics

To determine the time required to reach adsorption equilibrium, a fixed adsorbent dose was contacted with 30 mL of a 5 mg L^−1^ aqueous VFX solution, prepared in either ultrapure water or wastewater. Kinetic experiments were performed as described in Section 3.4.1, using an adsorbent dose of 50 mg L^−1^ for both materials and matrices. Each polypropylene tube containing the material suspension was shaken for varying contact times ranging from 5 to 240 min. The amount of VFX adsorbed at time *t* (*q*_t_, mg g^−1^) was calculated using Equation (3). Experimental data were fitted to the pseudo-first order model, the pseudo-second order model [51], and the intraparticle diffusion model [52], as depicted in Equations (4)–(6), respectively, to determine the kinetic parameters for each system:

(3) $$q_{t}=\frac{\left(C_{c}-C_{t}\right)V}{m}$$

(4) $$q_{t}=q_{e}(1-e^{-k_{1}t})$$

(5) $$q_{t}=\frac{q_{e}^{2}k_{2}t}{1+k_{2}q_{e}t}$$

(6) qt=kid×t12+C

where *C*_c_ (mg L^−1^) is the concentration of pharmaceutical in the corresponding control experiments (from now on, taken as the initial pharmaceutical concentration), *C_t_* (mg L^−1^) is the concentration of pharmaceutical in solution at time *t*, *V* (L) is the volume of solution, *m* (g) is the mass of adsorbent, *q_e_* (mg g^−1^) refers to the amount of adsorbate per unit mass of adsorbent at equilibrium, *k*_1_ (min^−1^) is the pseudo-first order rate constant, *k*_2_ (g mg^−1^ min^−1^) is the pseudo-second order rate constant, *k*_id_ is intraparticle diffusion rate constant (mg g^−1^ min^−1/2^) and *C* is intercept related to boundary-layer (film) effect. Non-linear fittings were performed using GraphPad Prism, version 8.0.2.

#### 3.4.3. Adsorption Equilibrium

For adsorption isotherm determination, solutions were shaken for 240 min to ensure equilibrium, while varying the adsorbent dose and keeping the initial VFX concentration constant. Briefly, aqueous VFX solutions (5 mg L^−1^), prepared in either ultrapure water or wastewater, were combined with varying amounts of each carbon adsorbent. In ultrapure water, the dose of SBG-AC-CP ranged from 15 to 125 mg L^−1^, whereas for SBG-AC-MP it ranged from 5 to 125 mg L^−1^; in wastewater, the range for SBG-AC-MP was 5 to 90 mg L^−1^. Adsorption equilibrium was not evaluated for SBG-AC-CP, as preliminary experiments required elevated doses of the material (over 200 mg L^−1^) to achieve reasonable removal percentages, making it ineffective.

At least eight different doses were tested for each system for the determination of the equilibrium adsorption capacity of the materials (*q*_e_, mg g^−1^), calculated according to Equation (7). Experimental data were fitted to the non-linear Langmuir and Freundlich models, expressed in Equations (8) and (9), respectively [53,54]:

(7) $$q_{e}=\frac{\left(C_{c}-C_{e}\right)V}{m}$$

(8) $$q_{e}=\frac{q_{m}\times K_{L}\times C_{e}}{1+K_{L}\times C_{e}}\;$$

(9) $$q_{e}=\;K_{F}\times C_{e}^{\left(\frac{1}{N}\right)}$$

where *C_e_* (mg L^−1^) is the concentration of VFX in the solution at the equilibrium; *q_m_* (mg g^−1^) is the Langmuir maximum adsorption capacity of each material towards VFX; *K_L_* (L mg^−1^) is the Langmuir equilibrium constant; K_F_ (mg g^−1^ (L mg^−1^)^1/N^) is the Freundlich equilibrium constant; *N* is the degree of nonlinearity; and all the other variables are defined as in Equations (4)–(6). Non-linear regression and parameter estimation for both models were performed using GraphPad Prism version 8.0.2.

### 3.5. Chromatographic Analyses

The remaining concentration of VFX in the aqueous phase after the adsorption experiments was determined by HPLC-FLD. Analyses were performed using a LC-20AD Prominence (model DGU-20A5) system equipped with a DGU-20A5 prominence degasser, an LC-20AD prominence high-pressure pump, and a CTO-10ASVP column oven all from Shimadzu (Japan), coupled to an ESA Inc. model 542 autosampler (Netherlands). Separation was achieved on an ACE5 C18 column (5 µm, 150 × 4.6 mm). The mobile phase consisted of acidified water with 0.37% (*v*/*v*) HCl and acetonitrile (60:40, *v*/*v*), delivered at a flow of 0.8 mL min^−1^. The injection volume was 20 µL, and the total run time was 7 min. Both the acidified water and acetonitrile were filtered by 0.22 μm membrane filters (Whatman, Cytiva, United Kingdom) before use. The column was conditioned before and after each batch analysis with 100% acetonitrile. VFX detection was performed with an RF-20A XS Prominence fluorescence detector (Shimadzu (Japan)) equipped with a Xe lamp, using an excitation wavelength of 230 nm and an emission wavelength of 298 nm [55].

The calibration curve was determined by analyzing standard solutions of VFX with concentrations ranging from 0.10 to 5.0 mg L^−1^, prepared in ultrapure water. Standards were filtered by 0.22 μm PVDF filters (Whatman) before injection. All standard solutions were analyzed in triplicate. Limits of detection (LOD) and quantification (LOQ) were calculated using Equations (10) and (11), respectively:

(10) $$\mathrm{LOD}\;=\;\frac{3.3\cdot \;\sigma }{b}$$

(11) $$\mathrm{LOQ}=\frac{10\cdot \sigma }{b}$$

where σ is the standard deviation of the intercept and b is the slope of the calibration curve [56].

Linearity was calculated by Equation (12):

(12) Lin(%) = 100 − RSD_b_

where RSD_b_ is the relative standard deviation of the curve’s slope (in percentage) [57].

### 3.6. Statistical Treatment of Data

Statistical comparison of adsorption data was carried out using a significance *t*-test at the 95% confidence level, based on three independent replicates, to assess differences for pre-defined pairs of conditions (e.g., comparing materials at the same dose and matrix, or comparing the same material in different matrices). Given that each comparison involved only two groups, *t*-tests were considered the most appropriate approach for this dataset.

## 4. Conclusions

Activated carbons derived from SBG were produced via conventional and microwave pyrolysis using K_2_CO_3_ activation to assess the effect of two pyrolysis approaches on adsorption performance towards VFX removal. The slightly superior textural characteristics of SBG-AC-MP indicate that microwave-assisted pyrolysis gives a better adsorbent material compared to conventional pyrolysis under the same activation conditions. Preliminary adsorption experiments showed high removal efficiencies in ultrapure water at low adsorbent doses (50 mg L^−1^). In wastewater, however, the performance diverged: SBG-AC-MP retained relatively high capacity, whereas SBG-AC-CP required higher doses to achieve comparable removals, demonstrating the higher robustness of SBG-AC-MP to matrix interferences.

Kinetic studies revealed that adsorption is best described by the pseudo-second-order model. Equilibrium was typically reached within ~2 h, with rapid uptake during the first 30 min due to abundant accessible active sites. The Langmuir isotherm provided the best fit to equilibrium data, consistent with monolayer adsorption on homogeneous surfaces. Maximum adsorption capacities followed the order: SBG-AC-MP (ultrapure water, 74 ± 6 mg g^−1^) > SBG-AC-MP (wastewater, 72 ± 2 mg g^−1^) > SBG-AC-CP (ultrapure water, 41 ± 1 mg g^−1^). Importantly, SBG-AC-MP in wastewater did not suffer a decay in performance, with no evidence of inhibitory matrix effects.

In conclusion, microwave-assisted pyrolysis yields high-performance adsorbents with superior physicochemical properties and adsorption behavior compared to conventional pyrolysis. This strategy enables the valorization of agro-industrial residues such as SBG, while simultaneously offering a sustainable and effective solution for removing pharmaceutical contaminants from aqueous environments. Future studies should focus on optimizing yields, evaluating regeneration potential, and extending applications to diverse contaminants and water matrices to support large-scale water treatment implementation.

## Figures and Tables

**Table 1:** Specific surface area (*S*_BET_) and textural parameters of the produced ACs (SBG-AC-CP and SBG-AC-MP), including total pore volume (*V_p_*), micropore volume (*W*_0_), average micropore width (*L*), and average pore diameter (*D*).

AC	N_2_ Adsorption at −196 °C				
	*S*_BET_ (m^2^ g^−1^)	*V_p_* (cm^3^ g^−1^)	*D* (nm)	Dubinin–Astakhov	
				*W*_0_ (cm^3^ g^−1^)	*L* (nm)
SBG-AC-CP	1080	0.48	0.88	0.43	1.47
SBG-AC-MP	1197	0.62	1.03	0.57	1.73

**Table 2:** Fitting parameters of the pseudo-first-order, pseudo-second-order, and intra-particle diffusion kinetic models applied to the experimental data for VFX adsorption onto SBG-AC-CP and SBG-AC-MP in ultrapure water and wastewater. *n* denotes the number of data points used for the fitting.

		Ultrapure Water		Wastewater
		SBG-AC-CP	SBG-AC-MP	SBG-AC-MP
Kinetic models				
Pseudo-first-order	*q_e_* (mg g^−1^)	30 ± 1	53 ± 2	64 ± 3
	*k*_1_ (min^−1^)	0.10 ± 0.02	0.05 ± 0.01	0.045 ± 0.008
	*r* ^2^	0.9537	0.9578	0.9600
	*n*	8	8	8
	*S_y/x_*	2.584	4.387	5.193
Pseudo-second-order	*q_e_* (mg g^−1^)	32.6 ± 0.8	58 ± 2	71 ± 2
	*k*_2_ (mg g^−1^ min)	0.0050 ± 0.0008	0.0012 ± 0.0002	0.0009 ± 0.0002
	*r* ^2^	0.9877	0.9844	0.9858
	*n*	8	8	8
	*S_y/x_*	1.332	2.663	3.092
Intra-particle diffusion model (Weber–Morris)	*k_id_*_(1)_ (mg g^−1^ min^1/2^)	3.0 ± 0.6	5.0 ± 0.1	6.1 ± 0.2
	*k_id_*_(2)_ (mg g^−1^ min^1/2^)	0.4 ± 0.3	1.0 ± 0.5	1.4 ± 0.5
	*C* _(1)_	10 ± 2	8.3 ± 0.5	10 ± 1
	*C* _(2)_	27 ± 3	40 ± 6	46 ± 6
	*R*^2^ _(1)_	0.9652	0.9994	0.9985
	*R*^2^ _(2)_	0.5087	0.7111	0.8076
	*S_y/x_*_(1)_	1.299	0.2996	0.5350
	*S_y/x_*_(2)_	1.519	2.658	2.836

(1) and (2) correspond to the fitting of the first and second stages in the Weber-Morris kinetic model, respectively.

**Table 3:** Operating conditions, adsorption kinetics, and isotherm parameters of ACs derived from SBG. The doses of adsorbates reported in the table refer to those used in the adsorption kinetic and isotherm tests. The doses of adsorbents reported in the table refer to those used in the adsorption kinetic tests.

Adsorbate and Concentration	Type of Pyrolysis	Activating Agent	*S*_BET_ m^2^ g^−1^	Adsorbent Dose (mg L^−1^)	Type of Matrix (pH)	*k*_1_ min^−1^	*r*^2^ PFO	*k*_2_ g·mg^−1^·min^−1^	*r*^2^ PSO	Best-Fit Model	*q_m_* mg g^−1^	Best-Fit Isotherm	r^2^	Reference
Tartrazine yellow dye (10 mg L^−1^)	Conventional	H_3_PO_4_/ SBG (1:1 ratio)	769	2000	Water adjusted (pH 3)	0.12	0.88	4.0·10^−2^	0.94	PSO	32	Langmuir	0.89	[44]
AMP (250 mg L^−1^)	Hydrothermal	KOH/ SBG (4:1 ratio)	1513	1000	Not specified	0.78	0.992	8.0·10^−3^	0.999	PSO	318	Langmuir	0.999	[45]
SMX TMP CFX (20 µmol L^−1^)	Microwave	K_2_CO_3_/ SBG (1:2 ratio)	929	15	Ultrapure water (pH 8)	0.11 0.04 0.21	0.959 0.973 0.974	3.6·10^−4^ 1.1·10^−4^ 14·10^−4^	0.992 0.987 0.939	PSO PSO PFO	120 244 116	Langmuir and Freundlich	0.995 0.970 0.990	[48]
CBZ (5 mg L^−1^)	Conventional	KOH/SBG NaOH/SBG H_3_PO_4_/SBG (1:1 ratio)	1120 267 29	20–40	Ultrapure water (no pH adjustment)	0.09 - -	0.959 - -	6·10^−4^ - -	0.985 - -	PSO - -	178 - -	Langmuir	0.966 - -	[48]
CBZ (300 mg L^−1^)	Hydrothermal	KOH/ NaCl (1:1 ratio)	906	1000	Water adjusted (pH 7)	190	0.971	1.0·10^−3^	0.995	PSO	190	Langmuir	0.976	[47]
VFX (5 mg L^−1^)	Microwave	K_2_CO_3_/ SBG (1:2 ratio)	1197	50	Ultrapure water (pH ~6)	0.05	0.958	1.2·10^−3^	0.984	PSO	74	Langmuir	0.973	Current work

The abbreviations AMP, SMX, TMP, CFX, and CBZ refer to “acetaminophen”, “sulfamethoxazole”, “trimethoprim”, “ciprofloxacin”, and “carbamazepine”, respectively. The abbreviations PFO and PSO stand for “pseudo-first-order” and “pseudo-second-order”, respectively.

**Table 4:** Fitting parameters of the Langmuir and the Freundlich equilibrium models for the adsorption of VFX onto SBG-AC-CP in ultrapure water, SBG-AC-MP in ultrapure water, and SBG-AC-MP in wastewater.

Equilibrium Models		Ultrapure Water		Wastewater
		SBG-AC-CP	SBG-AC-MP	SBG-AC-MP
Langmuir Freundlich	*q_m_* (mg g^−1^) *K_L_* (L mg^−1^) *r*^2^ *n* *S_y/x_* *K_F_* (mg g^−1^(mg L^−1^)^−N^) *N* *r*^2^ *n* *S_y/x_*	41 ± 1 7 ± 2 0.9907 8 1.394 36 ± 1 14 ± 7 0.9847 8 1.791	71 ± 4 2.0 ± 0.5 0.9725 7 4.048 46 ± 2 4 ± 1 0.9560 7 5.118	72 ± 3 2.1 ± 0.5 0.9843 9 2.671 50 ± 2 5 ± 1 0.9772 9 3.218

## Data Availability

Data is contained within the article and Supplementary Material.

## Supplementary Materials

The following supporting information can be downloaded from https://www.mdpi.com/article/10.3390/ph19030344/s1. This file contains a detailed description of the materials and methods used for carbon adsorbents characterization, namely point of zero charge (PZC), Specific surface area (*S*_BET_), and Scanning Electron Microscopy (SEM). Table S1. Structure and main physicochemical properties of Venlafaxine hydrochloride. Figure S1. ATR-FTIR spectra of the precursor SBG and the produced activated carbons (SBG-AC-CP and SBG-AC-MP) derived from it.

## Abbreviations

The following abbreviations are used in this manuscript:

AC	Activated Carbon
HPLC-FLD	High-Performance Liquid Chromatography with Fluorescence Detection
LOD	Limit of detection
LOQ	Limit of quantification
PZC	Point of Zero Charge
SBGs	Spent Brewery Grains
*S*_BET_	Specific surface area calculated using the method BET (Brunauer–Emmett–Teller)
SBG-AC-CP	Spent Brewery Grains-derived Activated Carbon produced via Conventional Pyrolysis
SBG-AC-MP	Spent Brewery Grains-derived Activated Carbon produced via Microwave Pyrolysis
SEM	Scanning Electron Microscopy
TOC	Total Organic Carbon
VFX	Venlafaxine
WWTPs	WasteWater Treatment Plants
