# Supplementary material for: Venlafaxine Removal from Water and Wastewater Using Activated Carbons from Spent Brewery Grains Produced by Conventional vs. Microwave Pyrolysis
Source: Pharmaceuticals (Basel). 2026 Feb 24;19(3):344. doi: 10.3390/ph19030344 (PMC13029031; doi:10.3390/ph19030344)
Supplement: Supplementary file 1 [file pharmaceuticals-19-00344-s001.zip › pharmaceuticals-4140273-supplementary.pdf]

SUPPLEMENTARY MATERIAL

Venlafaxine removal from water and wastewater using activated carbons from spent brewery grains produced by conventional vs. microwave pyrolysis

Angélica R. Zizzamia[1], Ângela Almeida[2], Maria V. Gil[3], Filomena Lelario[1], Vânia Calisto[2,*]

[1] Department of Basic and Applied Sciences, University of Basilicata, 85100 Potenza, Italy
[2] Department of Chemistry and CESAM, University of Aveiro, 3810-193, Aveiro, Portugal
[3] Instituto de Ciencia y Tecnología del Carbono, INCAR-CSIC, Francisco Pintado Fe 26, 33011 Oviedo, Spain

*Correspondence: vania.calisto@ua.pt

1. Chemical structure of venlafaxine

Table S1. Structure and main physicochemical properties of Venlafaxine hydrochloride.

**Molecular formula**	**Structure**	**Molecular weight (g mol^{-1})**	**Solubility in water (mg L^{-1})**	***pK$_a$***	**Log *K$_{ow}$***
$C_{17}H_{27}NO_2.HCl$	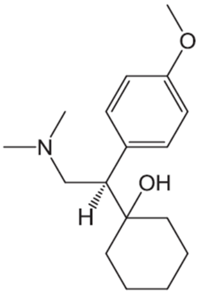 HCl	313.87	572000	8.91; 14.42	2.91

2. Carbon adsorbents characterization

2.1. Point of zero charge (PZC)

The PZC of SBG-AC-CP and SBG-AC-MP was determined using a batch equilibration method. A series of 0.1 M NaCl solutions with pH values ranging from 2 to 11 was prepared. The initial pH (pH_i) was adjusted using 0.1 M, 0.01 M, or 0.001 M HCl and 0.1 M, 0.01 M, or 0.001 M NaOH, while maintaining a constant ionic strength. Subsequently, 30 mL of each solution was transferred into polypropylene tubes containing either 1.5 mg of SBG-AC-CP or 1.5 mg of SBG-AC-MP (corresponding to a final dosage of 50 mg L^{-1}) and shaken at 80 rpm for 24 h. The final pH (pH_f) was then measured, and the PZC was determined by plotting ΔpH ($pH_f - pH_i$) versus pH_i. The PZC corresponds to the pH value at which the curve intercepts the x-axis ($pH_i = pH_f$).

2.2. Specific surface area (S_{BET})

S_{BET} of the produced materials was determined using a Micromeritics Gemini VII 2380 instrument (United States) at 77 K. Before analysis, the samples were degassed at 120 °C. Nitrogen adsorption–desorption measurements were performed using liquid nitrogen at −196 °C. The total pore volume (V_p) was estimated at a relative pressure of 0.99. The Brunauer–Emmett–Teller (BET) equation [55] was applied within a

relative pressure range of 0.001–0.1 to calculate S_{BET}. Microporosity (W_0) was evaluated from the low relative pressure region of the nitrogen adsorption isotherm using the Dubinin–Astakhov equation [56]. The average micropore width (L) was obtained from the Stoeckli–Ballerini equation [57]. The average pore diameter (D) was calculated according to the following equation:

$$D = 2x \frac{V_p}{S_{BET}}$$

2.3. Scanning Electron Microscopy (SEM)

SEM images were obtained at magnifications of 700×, 15,000×, and 35,000×. A Hitachi SU70 scanning electron microscope (Japan), operated at 1, 3, and 30 kV, was employed to capture micrographs of the microstructure of the produced materials. Before analysis, the samples were dispersed onto a thin carbon film using a carbon rod coater (Emitech K950X).

2.4. Fourier transform infrared spectroscopy with attenuated total reflectance (FTIR-ATR)

The characterization of the adsorbents included the qualitative analysis of the carbon functional groups, represented in Figure S1. The FTIR–ATR (Fourier transform infrared spectroscopy with attenuated total reflectance) spectra were obtained through a Bruker Alpha Platinum (Germany) equipped with a single reflection diamond ATR module with a resolution of 4 cm^{-1} from 400 to 4000 cm^{-1}, 64 scans, and with atmosphere and background correction.

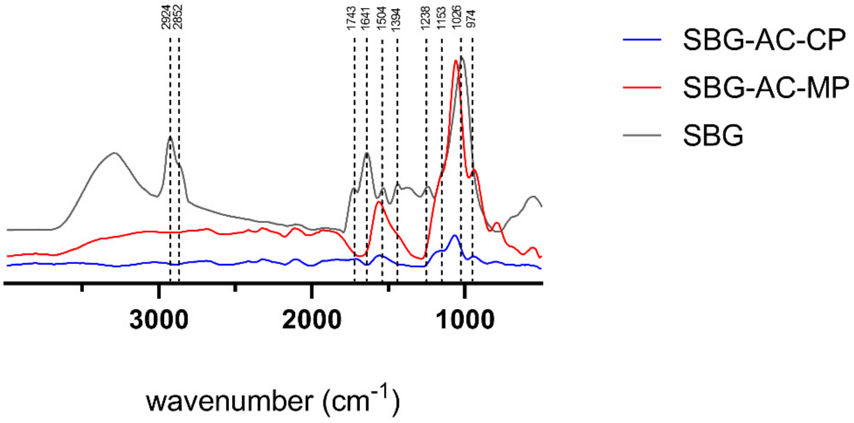

Figure S1. ATR-FTIR spectra of the precursor SBG and the produced activated carbons (SBG-AC-CP and SBG-AC-MP) derived from it.

References

55. Brunauer, S.; Emmett, P.H.; Teller, E. Adsorption of gases in multimolecular layers. J. Am. Chem. Soc. 1938, 60, 309–319. https://doi.org/10.1021/ja01269a023

56. Dubinin, M.M. Physical adsorption of gases and vapors in micropores. In Progress in Surface and Membrane Science; Academic Press: New York, NY, USA, 1975; Vol. 9, pp. 1–70. https://doi.org/10.1016/B978-0-12-571809-7.50006-1

57. Stoeckli, F.; Ballerini, L. Evolution of microporosity during activation of carbon. Fuel 1991, 70, 557–559. https://doi.org/10.1016/0016-2361(91)90036-A
